# The benefits of using atypical presentations and rare diseases in problem-based learning in undergraduate medical education

**DOI:** 10.1186/s12909-023-04079-6

**Published:** 2023-02-06

**Authors:** Suyun Bai, Limin Zhang, Zhen Ye, Duxiao Yang, Tao Wang, Yuanying Zhang

**Affiliations:** 1 Department of Molecular Biology and Biochemistry, School of Clinical and Basic Medical Sciences, Shandong First Medical University & Shandong Academy of Medical Sciences, Jinan, China; 2 Department of Rheumatology and Immunology, Shandong Provincial Hospital Affiliated to Shandong First Medical University, Jinan, China

**Keywords:** PBL, Case scenarios, Rare disease, Undergraduate medical education

## Abstract

**Background:**

The nature of student learning in problem-based learning (PBL) largely depends on the quality of the case scenarios presented to them. The effect of case scenarios with higher challenge degree, especially common disease with atypical symptoms (CDAS)- and rare disease (RD)-based case scenarios, on undergraduate medical students remains unclear. This study compared the impact of all scenarios pertaining to common disease with typical symptoms (CDTS) case scenarios, CDTS interspersed with CDAS case scenarios, and CDTS interspersed with RD case scenarios on perceptions of undergraduate students studying organ/system integration curriculum via PBL.

**Methods:**

After finishing four CDTS case scenarios, 294 third-year medical students were randomly allocated into three groups: CDTS, CDAS and RD, studying via CDTS, CDAS and RD case scenarios, respectively. A questionnaire with 15 items was conducted to evaluate the students’ perceptions. The students’ responses were scored using a 4-point rating scale. The data were analysed using the Kruskal–Wallis test.

**Results:**

Among the three PBL conditions, the ones with a higher degree of challenge were rated higher by the students, which included the quality of the case scenarios and the overall performances of the students. The CDAS and RD cases were more effective in developing students’ self-directed learning skills, stimulating them to acquire more knowledge required for future work. The satisfaction percentage of RD case scenario sessions was higher.

**Conclusions:**

Of all the three kinds of case scenarios, both CDTS interspersed with CDAS and RD case scenarios had more positive effects on the self-evaluated performance of students. Increasing the challenge and variety of case scenarios by the inclusion of CDAS and RD especially RD might be an effective stimulus in improving students’ performance in PBL sessions.

**Supplementary Information:**

The online version contains supplementary material available at 10.1186/s12909-023-04079-6.

## Background

In the late 1960s, the newly established medical school of McMaster University in Canada introduced problem-based learning (PBL) as a novel teaching concept, which has gained much attention in recent years in medical education worldwide. It advocates ‘student-centred, competency-oriented, case scenario-based [curriculum that also focuses on] group discussion mode’, and concentrates on stimulating students’ interest in self-learning by working collaboratively, cultivating their communication skills, and strengthening critical and innovative thinking [1, 2]. Although PBL has been widely adopted in medical education, its effectiveness is still debated, particularly in Asian medical colleges [3, 4]. Kwan [5] even claimed that despite being popular across medical schools in Asian countries, the implementation of PBL failed due to misconceptions and distortions of its concept. Shimizu [2] indicated that Asian educators have struggled to implement PBL as students rarely discuss their work actively and are not sufficiently engaged in self-directed learning. These reflected the methodological and conceptual understanding difficulties encountered. When PBL is implemented, educators should pay attention to students’ prior knowledge and stimulation of self-directed learning [6]. It is well-known that the case scenario is the soul of PBL. The nature of learning in PBL depends largely on the quality of the case scenarios presented to them [7–9]. Therefore, it is necessary that case scenarios be developed and updated continuously.

Problem-based learning in the undergraduate MD program at McMaster University focuses on priority health problems, and centres on a list of common medical problems as the foundation for curriculum organisation, on the basis that an understanding of the management of common conditions includes areas of knowledge that would be essential for clinical competence [10]. PBL case scenarios in the medical undergraduate program of China, including our university, are often designed as common case scenarios [9]. Moreover, in most medical universities or colleges of China (including our university), the teaching programs mainly follow a traditional didactic model, with online and offline blended teaching model increasing gradually in recent years. PBL still accounts for a small proportion due to limited faculty resources, but because of its unique advantages, all colleges and universities attach great importance to it. As an educational method, PBL should stay abreast of the changes of national conditions, school conditions, and learning conditions and be continuously developed through continuous evaluations and feedback [11]. In recent years, China has begun to pay increasing attention to the prevention and early diagnosis and treatment of diseases, especially rare diseases that had been ignored for a long time. In addition, the ‘Construction of China’s “Golden Courses”’ program places higher demands on teachers and students. We hypothesise that the inclusion of common diseases with atypical symptoms (CDAS) and rare diseases (RD) in typical PBL case scenarios in order to increase the challenge to students and enrich the variety of cases would be an effective stimulus in improving students’ learning skills. The major object of this study was to evaluate the learning outcomes of students involved in three PBL case scenario conditions: all CDTS, CDTS interspersed with CDAS, and CDTS interspersed with RD.

## Materials and methods

### Setting

In the last century, integration of the curriculum including organ-system and problem-based curricula began to be adopted in medical education. Integrated teaching was first introduced at the Case Western Reserve University Medical School in 1952 in one course [12]. The curriculum combines independent disciplines in an integrated approach, usually organized around an organ/system of the body. In 1985, Harvard Medical School adopted a "New Pathway" curriculum, which is a successful example of the integration of the integrated curriculum with PBL [13]. This reform of medical education is a "revolutionary" transformation and innovation. In 1997, the integration of the integrated curriculum with PBL was launched in University of Hong Kong [14]. Now the integration of the integrated curriculum with PBL mode has become a well-accepted and widely used teaching mode in many medical schools all over the world. The integration of medical courses in China is still in the exploratory stage. A study of 15 medical colleges and universities in China showed that 30% of the institutions adopt a completely traditional discipline-based curriculum model, and the left 70% adopt traditional discipline-based curriculum and organ/system integration curriculum hybrid teaching model [15]. The systematic integrated teaching is carried out by various teaching methods including PBL, case-based learning (CBL) and team-based learning (TBL), among which PBL is adopted by all the above mentioned 70% institutions [15]. The hybrid teaching model of traditional discipline-based curriculum and organ/system integration curriculum is also adopted by our university.

### Study design and participants

Before organ/system integration curriculum PBL sessions, students have received general education and basic medical science education including medical ethics, clinical communication, epidemiology, evidence-based medicine, systematic anatomy, histology, embryogenesis, cell biology, topical anatomy, physiology, biochemistry, immunology, pharmacology, pathology, and pathophysiology. Most courses follow the traditional large class didactic model or are interspersed with online and offline hybrid teaching. Thereafter, to optimize the transition between the basic medical science curriculum and the clinical curriculum, in the second semester of the third year, students enter the organ/system integration curriculum PBL and clinical science learning stage. In this semester, they will learn 10 PBL case scenarios of different specific organ system units including the endocrine and reproductive system, infections and immune system, urinary system, hematologic system, cardiovascular system, respiratory system, mental disorders including severe cases, digestive system, and neurological system. Under the guidance of facilitators, students complete each case scenario in two discussion sessions (three hours/session) spanning over two weeks.

For this study, 294 third-year clinical medicine undergraduates were enrolled. At the beginning, as they were unfamiliar with this kind of teaching mode, they were offered CDTS case scenarios. Thereafter, in the midterm of PBL learning semester, namely after four case scenarios, all students were randomly assigned into three age and sex-matched groups: the CDTS group studied via CDTS cases, the CDAS group studied via CDAS cases, and the RD group studied via RD cases, comprising 100, 98, and 96 students, respectively. Students of each group were then further randomly divided into 9 sub-groups with about 11 students per sub-group. Six case scenarios were chosen. Although the CDAS and RD case scenarios are uncommon, but they are also helpful to study key knowledge related to CDTS. The case scenarios were modified from real cases by a same trained PBL writing team to suit the stage of the curriculum and the students’ learning needs. During the construction of the PBL case scenarios, the members in the team used a same PBL template, constructed tutor’s guides and collected required images. All the case scenarios were validated by the clinical experts and approved by PBL case scenario experts of our college. Typical acute B lymphoblastic leukemia, atypical acute B lymphoblastic leukemia and paroxysmal nocturnal hemoglobinuria (PNH) of the hematologic system were the first batch of CDTS, CDAS and RD case scenarios assigned to the three groups, respectively. The second batch of CDTS, CDAS and RD case scenarios was typical hepatic encephalopathy, atypical hepatic encephalopathy and hepatolenticular degeneration of the digestive system. All teaching processes of the three groups were accomplished by the same teaching faculties. Relevant resource materials were available for all students, including textbooks, journal articles, and Internet sources to gather information regarding the problem presented by the case. After the PBL sessions of the 6 case scenarios, a survey was conducted via an anonymous online questionnaire with open-ended questions.

### The assessments of student perceptions

Taking cue from previous studies [9, 16–18], we modified and developed a 15-item open-ended online survey to evaluate student perceptions of the effectiveness of the case scenario sessions.

The associated survey items were categorised into two areas: students’ attitudes towards the quality of cases and their self-evaluation of the skills and content of case scenario sessions. There was an additional space for comments on the advantages and disadvantages of RD case scenario sessions. Evaluations were completed by students immediately at the end of the 6 case scenario sessions. Respondents scored survey items on a 4-point rating scale, with 0 = *strongly disagree or disagree*, 1 = *neutral*, 2 = *agree*, and 3 = *strongly agree*.

### Statistical analysis

The items were analysed using the Kruskal–Wallis test. Furthermore, SPSS for Windows, version 25 (IBM SPSS), was used for all analyses.

## Results

### Student assessments and perceptions

All students participated in the questionnaire survey. Table 1 shows students’ attitudes towards the challenge degree of the three types of case scenarios. Students felt that the latter two types of case scenarios, based on CDAS and RD, were more difficult than the first type based on CDTS (*p* < 0.001). Moreover, with the increasing of the challenge degree, although a few students (2.08%) felt that RD cases were too difficult and not suitable for them, a majority of the students (66.20% for the CDAS group and 77.08% for the RD group) felt that the cases provided to them were difficult, but they preferred difficult cases. There was no difference of challenge degree between the CDAS and RD case scenarios (*p* > 0.05).

**Table 1:** Students’ attitudes towards the challenge degree of the three types of cases

Challenge degree of cases	CDTS, *n* (%)	CDAS, *n* (%)	RD, *n* (%)
Too easy and learned too little	6 (6)	0 (0)	0 (0)
Medium challenge degree	59 (59)	39 (39.80)	20 (20.83)
Difficult, but I like difficult cases	35(35)	59 (60.20)	74 (77.08
Too difficult and not suitable for me	0 (0)	0 (0)	2 (2.08)

Analysed by means of the Kruskal–Wallis test

The *H*-values of the CDTS, CDAS, and RD groups are 111.83, 151.59, and 180.48, respectively

^**^p < 0.001, CDTS vs CDAS vs RD

^*^p < 0.001, CDTS vs CDAS

^***^p < 0.001, CDTS vs RD

*p* > 0.05, CDAS vs RD

As presented in Table 1 (Additional file 1), no difference in interest was noted between the three types of case scenarios. Compared to CDTS case scenarios, both CDAS and RD were rated more authentic and logical (*p* < 0.05); CDAS case scenarios were more beneficial in identifying and achieving the learning objectives (*p* < 0.05), improving the ability to collect, reorganise, and analyse information and for critical thinking abilities (*p* < 0.01); and RD involved more medical humanities, healthcare, and disease prevention (*p* < 0.001). Furthermore, compared to CDTS, RD case scenarios were especially beneficial in promoting the integration of knowledge and learning the frontiers of knowledge (*p* < 0.001) and in improving the ability to collect, reorganise, and analyse information and critical thinking abilities (*p* < 0.01).

Table 2 (Additional file 1) depicts students’ self-evaluation on skills and the content of the case scenario sessions in the three PBL groups. As shown in this table, students were more satisfied with the latter two types of case scenarios. They felt that both CDAS and RD case scenarios were especially beneficial to their study and work in the future than CDTS case scenarios (CDTS vs CDAS: *p* < 0.05, CDTS vs RD: *p* < 0.01). CDAS case scenarios were more helpful for developing deeper self-awareness than CDTS (*p* < 0.05). Furthermore, compared to CDTS, RD case scenarios were superior in helping them develop their skills including clinical thinking skills (*p* = 0.01), interpersonal skills including navigating a team with various personalities, working with challenging personalities, and physician–patient communication (*p* < 0.01), deeper self-awareness (*p* < 0.01), and professionalism (*p* < 0.01). Students were more satisfied with the RD case scenario sessions (CDTS vs RD: *p* = 0.01). However, regarding improving presentation skills and leadership, no differences between the three groups were noted.

### Students’ comments on the value of CDAS and RD case scenario sessions

Although a few (2.08%) students in the RD group complained that they spent more time on information retrieval and discussion among group members after classes, most of them expressed positive opinions on the CDAS and RD case scenario sessions. Detailed below are some examples of verbatim quotes (the student number is given in brackets):

‘Compared to the previous case scenarios, these case scenarios are more informative’. (N9 in RD group and N15 in CDAS group).

‘The cases are novel, interesting, [and] challenging. They involve the doctor-patient relationship and integrate population, behavior, and life science more effectively’. (N11 in RD group).

‘The cases are more authentic and logical’. (N10 in RD group).

‘Rare diseases are deeply rooted in our hearts’. (N22 in RD group).

‘The cases are more helpful for divergent thinking. Moreover, they guide us to discuss more about basic medical institutions and our country’s medical system’. (N78 in RD group).

‘ [The use of] RD [-based scenarios in our curriculum] broadened the scope of my knowledge. Our thinking has been limited to common diseases and ignored rare diseases [earlier]’. (N5 in RD group).

‘We students were more active and more involved in the sessions’. (N18 in RD group and N2 in CDAS group).

‘The cases were novel, real and stimulated our curiosity’ (N5 in CDAS group).

‘These case scenario sessions required more teamwork, and they improved our ability to think comprehensively’ (N30 in CDAS group).

After our RD sessions, soon came a rare disease awareness day (for osteogenesis imperfecta). Students were actively involved in awareness-raising campaigns on rare diseases. Responses from the students showed that this further deepened their awareness and concern about rare diseases.

## Discussion

There is a dearth of research aiming at investigating the effects of the challenge degree of case scenarios, especially CDAS- and RD-based case scenarios, on the effectiveness of PBL. In this study, we found that as PBL sessions progressed, after students were accustomed to PBL based on CDTS cases, they showed greater satisfaction with the challenging CDAS and RD cases, especially the latter, which integrated more PBL philosophy of population and behaviour. The CDAS and RD cases were more effective in improving their self-evaluated skills and knowledge acquirement required for future work. Making students engaging is an important key for successful PBL cases. There is evidence that engagement occurs when students find that the case: (a) relates to their learning needs, (b) builds on what they learnt from previous cases, (c) encourages their interaction and thinking processes, (d) relates to their life and feelings and touches on psychosocial/moral issues, (f) gives some challenging situations, (g) gives students a sense of satisfaction and a learning experience as they complete the case discussion [19]. According to students’ attitudes towards the three types of cases and comments on the value of RD and CDAS case scenario sessions, for students having accustomed to CDTS cases, both CDAS and RD cases especially RD were more in line with the above criteria than CDTS.

Jie Li et al. [9] claimed that the failure of PBL implementation in Asian countries might be explained by the following factors: (1) Students in China had grown accustomed to traditional didactic teaching, which might have changed the learning style dramatically, affecting their perceptions and learning outcomes; (2) student learning outcomes in PBL might depend on specific features of the subject itself; (3) the existence of a sampling bias resulting from small sample sizes [9]. As with other teaching styles, PBL’s effectiveness is determined by national conditions, the school situation, and the learning situation. In recent years, education in China has gradually changed from traditional didactic teaching to student-centred teaching. The factors that determine the teaching effect of PBL have changed accordingly with time.

In November 2018, at the 11^th^ Chinese University Teaching Forum, Wu Yan, director of Higher Education Division of Ministry of Education, delivered a report titled ‘Construction of Chinese “Golden Courses”’. Yan proposed the standards of ‘golden courses’, namely ‘high order’, ‘innovation’, and ‘challenge degree’, where ‘high order’ implies the organic integration of knowledge, ability, and quality, with the intention of cultivating students’ comprehensive quality to solve complex problems and high order thinking ability. ‘Innovation’ implies that the course content is contemporary, the teaching form is advanced and interactive, and the learning results are exploratory and individual. ‘Challenge degree’ means that the course should be of certain difficulty, making higher demands on teachers and students [20]. The implementation of the ‘Construction of Chinese “Golden Courses”’ in the curriculum has considerably improved the ability and quality of the students which are also cultivated in PBL, including clinical thinking, presentation skills, interpersonal skills, solving complex problems, leadership and so on. Therefore, ‘Construction of Chinese “Golden Courses”’ may be helpful for students' adaptation to PBL learning style and lay the foundation for learning challenging RD and CDAS.

Rare diseases (RDs) are diseases with very low incidence and prevalence rates. However, due to the huge population of China, it is estimated that there are at least 16.8 million patients afflicted with rare diseases in China, representing a significant challenge for the whole healthcare system that should not be neglected [21]. Difficulty in diagnosis and treatment are common embarrassing situations for rare disease patients worldwide [22]. A recent survey of 224 physicians from different hospitals in China showed that, although most physicians (83.5%) were from tertiary hospitals, only 5.3% of physicians were moderately or well aware of rare diseases [21]. Moreover, 78.8% of RD patients believed that they could not receive proper care due to the lack of awareness and knowledge among physicians [21]. Therefore, Rare diseases cause both medical and social problems. Improving the diagnosis and treatment of RDs is not merely an urgent need to guarantee the health and life rights of patients, but also an inevitable requirement to realise national unity, equity, and justice [23]. Due to the lack of rare disease teaching in previous study, students knew little about ‘population’, ‘behavior’ and ‘life science’ in the field. In our RD PBL sessions, students paid much attention to RD humanistic care, population health and health policies. Therefore, in the RD sessions, the essence of PBL could be studied more deeply and comprehensively, which was more conducive to the cultivation of students’ comprehensive quality. 

The early and atypical presentations of CDAS cases are the common factors that frequently lead to missed diagnosis, misdiagnosis, or late diagnosis. Three common conditions including cancers, vascular events and infections are the ‘Big Three’ causes of diagnostic errors, accounting ~ 75% of serious harms from diagnostic errors [24]. The positive results of our studies indicated that both CDAS or RD-based case scenarios might offer a way in to reduce the diagnostic errors in the students’ future clinical practice.

It seemed that CDAS and RD made students more cautious and think more comprehensively. For example, after CDAS and RD sessions, many students expressed their feelings “Don't make a diagnosis in seconds, because you can only see what you can see, and you can't find what you don’t know! For cultivation of clinical reasoning, there’s a long way to go.” However, this also reflected some potential risks in teaching students about RD, as it can skew their diagnostic reasoning for CDTS in the future clinical practice, thus hinder their timely and economical correct diagnosis of CDTS, which have higher morbidity and mortality. Therefore, when CDAS and RD are included in PBL of medical undergraduates especially those with limited clinical experience, educators should carefully design the case scenarios and guide the discussions in the sessions, making the students focus on common diseases and pay proper attention to CDAS and RD. Namely, in diagnosing diseases, they must give more consideration to common diseases with higher incidence.

### Limitations

Our study has several limitations that should be noted. First, our study is limited to students’ immediate subjective feedback, lacking long-term objective supporting data, such as the differences in students’ future performance in clinical practice. Second, this study is only a preliminary investigation on the role of CDTS and RD case scenarios in PBL. In the future, more comprehensive and detailed studies should be conducted, including the more precise appropriate timing of CDAS and RD case scenarios in the PBL semester, the proportion of CDAS and RD case scenarios in all case scenarios, the selection of related diseases, case scenario writing skills, etc. All these factors may have an impact on the PBL effect.

## Conclusions

Of all the three types of case scenarios, both CDTS interspersed with CDAS and RD case scenarios were preferred by students. Overall, students’ satisfaction percentage with RD sessions was higher. Furthermore, the inclusion of RD in PBL is also an urgent need for the construction of social security systems that guarantee national unity, equity, and justice. Therefore, after students have become familiar with CDTS PBL sessions, a certain percentage of CDAS and RD case scenarios might be an effective stimulus in improving students’ performance in PBL sessions.

## Supplementary Information

**Additional file 1: Table 1.** Comparison of students’ attitudes towards the quality of the cases in the three groups (mean±SD). **Table 2.** Comparison of students’ self-evaluation in the three PBL groups (mean±SD).

## Data Availability

The datasets used and/or analysed during the current study are available from the corresponding author upon reasonable request.

## Abbreviations

PBL: Problem-based learning
CDAS: Common disease with atypical symptoms
RD: Rare disease
CDTS: Common disease with typical symptoms
