# Supplementary material for: The benefits of using atypical presentations and rare diseases in problem-based learning in undergraduate medical education
Source: BMC Med Educ. 2023 Feb 6;23:93. doi: 10.1186/s12909-023-04079-6 (PMC9900952; doi:10.1186/s12909-023-04079-6)
Supplement: Supplementary file 1 — Additional file 1: Table 1. Comparison of students’ attitudes towards the quality of the cases in the three groups (mean±SD). Table 2. Comparison of students’ self-evaluation in the three PBL groups (mean±SD). [file 12909_2023_4079_MOESM1_ESM.docx]

**Table 1.** Comparison of students’ attitudes towards the quality of the cases in the three groups (mean±SD)

| **Items CDTS CDAS RD *H*-Value *P*-Value^a^  *P*-Value^b^ *P*-Value^c^ *P*-Value^d^** |
| --- |
| 1. The cases were very interesting 1.92±0.85 2.12±0.69 2.18±0.73 5.26 > 0.05  2. The cases were authentic and logical 2.44±0.61 2.63±0.48 2.57±0.58 6.04 < 0.05 < 0.05 < 0.05 > 0.05  3. Being able to identify and achieve learning objectives 2.50±0.61 2.69±0.51 2.66±0.52 6.25 < 0.05 < 0.05 > 0.05 > 0.05  4. Medical humanities, health care, and disease prevention were 2.44±0.70 2.61±0.60 2.78±0.42 16.7 < 0.001 > 0.05 < 0.001 < 0.05  involved in the cases  5. Being helpful to promote the integration of 2.52±0.58 2.63±0.53 2.70±0.46 6.87 < 0.05 > 0.05 < 0.001 > 0.05  knowledge and learn about the frontiers of knowledge  6. Greatly improved the ability to collect, re-organise, and 2.52±0.54 2.69±0.55 2.70±0.51 11.13 < 0.01 < 0.01 < 0.01 > 0.05  analyse information and critical thinking abilities |

Notes: Analysed with the Kruskal–Wallis test.

^a^Compared between the three PBL groups

^b^Compared between CDTS and CDAS groups

^c^Compared between CDTS and RD groups

^d^Compared between CDAS and RD groups

**Table 2.** Comparison of students’ self-evaluation in the three PBL groups (mean±SD)

| **Items CDTS CDAS RD *H*-Value *P*-Value^a^  *P*-Value^b^ *P*-Value^c^ *P*-Value^d^** |
| --- |
| 1. Beneficial to the study and work in the future 2.07±0.78 2.32±0.74 2.40±0.71 7.50 < 0.05 < 0.05 < 0.01 > 0.05  2. Beneficial to cultivate clinical thinking 2.54±0.58 2.63±0.60 2.71±0.58 6.76 < 0.05 > 0.05 = 0.01 > 0.05  3. Beneficial to improve presentation skills 2.49±0.69 2.57±0.67 2.67±0.59 4.23 > 0.05  4. Beneficial to cultivate interpersonal skills including 2.38±0.71 2.59±0.53 2.63±0.60 7.73 < 0.05 > 0.05 < 0.01 > 0.05  navigating a team with diverse personalities, working  with challenging personalities, and physician-patient  communication  5. Beneficial to develop deeper self-awareness 2.46±0.58 2.57±0.64 2.67±0.59 8.87 < 0.05 < 0.05 < 0.01 > 0.05  6. Beneficial to develop professionalism 2.39±0.72 2.57±0.61 2.69±0.47 8.61 < 0.05 > 0.05 < 0.01 > 0.05  7. Beneficial to develop leadership 2.36±0.70 2.45±0.73 2.48±0.74 2.55 > 0.05  8. Overall, I was satisfied with the case scenario sessions 2.19±0.83 2.40±0.76 2.48±0.68 7.22 < 0.05 > 0.05 = 0.01 > 0.05 |

Notes: Analysed with the Kruskal–Wallis test.

^a^Compared between the three PBL groups

^b^Compared between CDTS and CDAS groups

^c^Compared between CDTS and RD groups

^d^Compared between CDAS and RD groups
